# Juvenile‐onset motor polyneuropathy in Siberian cats

**DOI:** 10.1111/jvim.15963

**Published:** 2020-11-11

**Authors:** Kelly C. Crawford, Dayna L. Dreger, G. Diane Shelton, Kari J. Ekenstedt, Melissa J. Lewis

**Affiliations:** ^1^ Department of Veterinary Clinical Sciences College of Veterinary Medicine, Purdue University West Lafayette Indiana USA; ^2^ Department of Basic Medical Sciences College of Veterinary Medicine, Purdue University West Lafayette Indiana USA; ^3^ Department of Pathology School of Medicine, University of California San Diego La Jolla California USA; ^4^ Present address: VCA Alameda East Denver Colorado USA; ^5^ Present address: National Human Genome Research Institute, Cancer Genetics and Comparative Genomics Branch, National Institutes of Health Bethesda Maryland USA

**Keywords:** feline, inherited, muscle, neuromuscular, pedigree, peripheral nerve, weakness

## Abstract

**Background:**

Polyneuropathies are infrequently described in cats. There is a genetic predisposition in several breeds.

**Objective:**

To clinically characterize a novel motor polyneuropathy in a family of Siberian cats.

**Animals:**

Thirteen closely related Siberian cats, 4 clinically affected and 9 clinically unaffected individuals.

**Methods:**

Retrospective study. Clinical data and pedigree information were obtained from the medical records and breeder. Electrodiagnostic testing and muscle and peripheral nerve biopsy samples were obtained from 1 affected cat. Follow‐up information was obtained for all affected cats.

**Results:**

Onset of signs was 4 to 10 months in affected cats. Clinical signs were progressive or waxing/waning neuromuscular weakness (4/4), normal sensory function (4/4), and variably decreased withdrawal reflexes (3/4). All cats returned to normal neurologic function within 1 to 4 weeks. All cats had a recurrence of weakness (3/4 had 1 recurrent episode, 1/4 had 3 relapses) from which they recovered fully. In 1 cat, electromyography and motor nerve conduction studies showed multicentric spontaneous activity, normal motor nerve conduction velocity, reduced compound muscle action potential amplitude, and polyphasia. Histologic evaluation of muscle and nerve in that cat showed mild muscle atrophy consistent with recent denervation, endoneurial and perineurial edema, and mild mononuclear cell infiltration within intramuscular nerve branches and a peripheral nerve. Pedigree analysis suggests an autosomal recessive mode of inheritance, although neither a genetically complex/polygenic condition nor an acquired inflammatory polyneuropathy can be ruled‐out.

**Conclusions and Clinical Importance:**

We describe a motor polyneuropathy in juvenile Siberian cats characterized by self‐limiting weakness with potential relapse.

## 1 INTRODUCTION

Acquired and congenital or inherited polyneuropathies are infrequently described in cats.^1^, ^2^, ^3^ Acquired forms are attributed to infectious agents, intoxications, metabolic, immune‐mediated, inflammatory, neoplastic, or paraneoplastic conditions, although a definitive cause is not identified in most cases.^1^, ^3^, ^4^, ^5^, ^6^, ^7^, ^8^, ^9^, ^10^, ^11^, ^12^, ^13^ There is a genetic predisposition to disease affecting young cats of several breeds including Snowshoe, Abyssinian, and Bengal cats.^2^, ^14^, ^15^, ^16^, ^17^

Clinical presentations among the published cases are variable, but consistent findings include progressive neuromuscular weakness in young animals with potential for relapse of signs and no consistent response to, or recommendation for, specific therapy.^7^, ^9^, ^14^, ^16^, ^17^, ^18^ Electrodiagnostic testing and nerve and muscle biopsies are intermittently performed with variable results between specific reports. In the largest published case series, which described affected Bengal cats, findings are consistent with recurrent demyelination and remyelination as the pathophysiological underpinning of clinical signs of weakness and the chronic relapsing course of disease.^14^ However, the underlying cause of neuropathy in Bengal cats has not been identified. A motor neuropathy of unknown cause is described in a group of 5 unrelated cats of various breeds.^18^ While all cats had a juvenile onset and potential for recurrence or progression of signs, the heterogeneity in breed, presentation, and diagnostic findings suggest there might be multiple etiologies.^18^ Among the published reports, many cases are described as having self‐limiting signs but with the possibility of recurrence. Various specific treatments are employed including nonsteroidal anti‐inflammatories or corticosteroids, but evidence is lacking on the efficacy or role for such medications with these conditions. For those cats in which a hereditary component is suspected, an underlying genetic variant has not been identified.

We recently noted a progressive or waxing and waning neuromuscular weakness in several related Siberian Forest cats which share some clinical characteristics with the motor polyneuropathy of young cats. To the best of the authors' knowledge, a polyneuropathy has not been previously reported in this breed. Our objective was to characterize a novel motor polyneuropathy in a family of related Siberian cats.

## 2 MATERIALS AND METHODS

### 2.1 Case selection

Medical records of 4 related juvenile Siberian cats that showed clinical signs consistent with neuromuscular disease between April 2018 and March 2019 were available for review. All cats were from the same breeder. One cat was presented to the Purdue University College of Veterinary Medicine Veterinary Teaching Hospital and underwent thorough diagnostic evaluation as outlined below. Three additional affected cats were evaluated by a board‐certified veterinary neurologist or referring veterinarian.

Nine Siberian cats related to the 4 affected cats, and with no history of clinical signs of weakness were included as controls. Owners completed health questionnaires asking about overall health across body systems to confirm the health status of the control cats; referring veterinarian records were reviewed for additional information as available. Information on other related cats (eg, littermates or parents of control or affected cats used in the pedigree) was obtained from health questionnaires, which also asked about the general health status of related animals, if known.

### 2.2 Clinical and diagnostic evaluation

All available clinical records were reviewed, and results of physical and neurological examinations were analyzed. Results of additional diagnostic evaluation, such as hematology, serum biochemistry, urinalysis, creatine kinase activity, feline retroviral testing, other infectious disease testing, and diagnostic imaging, including thoracic and spinal radiographs, were reviewed when available. Any treatments administered were recorded and follow‐up information regarding clinical outcome was obtained from the owner, breeder, or veterinarian as available.

One cat underwent a full diagnostic work‐up for neuromuscular disease, including cerebrospinal fluid analysis, electrodiagnostic testing, acetylcholine receptor antibody titer, and muscle and peripheral nerve biopsy. Electrodiagnostic evaluation was performed under general anesthesia using a Cadwell Sierra Summit 4‐channel electrodiagnostic unit (Cadwell Industries, Inc, Kennewick, Washington). The cat was premedicated with .01 mg/kg IV acepromazine (acepromazine maleate 10 mg/mL, Vet One MWI Animal Health, Boise, Idaho) and .2 mg/kg IV methadone (methodone hydrochloride 10 mg/mL, Akorn Inc, Lake Forest, Illinois), induced with 2 mg/kg IV alfaxalone (Alfaxan Multidose, Jurox Animal Health, Kansas City, Missouri) and .2 mg/kg IV midazolam (midazolam hydrochloride 1 mg/mL, Alvogen Inc, Pine Brook, New Jersey), and then maintained under inhalant anesthesia with 1% to 2% isoflurane in oxygen. Body temperature was monitored and maintained between 37.0 and 37.8°C throughout the procedures. Electromyography was performed on multiple cranial, epaxial, and appendicular muscles in standard fashion using a concentric needle electrode and subcutaneous ground electrode. Insertional and any spontaneous activity were recorded for each muscle examined and scored using a published scoring system.^19^

Motor nerve conduction studies using monopolar stainless steel electrodes were performed on the ulnar and sciatic/tibial nerves according to standard protocol.^20^, ^21^, ^22^ For the ulnar nerve, stimulation was performed at the elbow and carpus with recording from the palmar interosseus muscles. For the sciatic/tibial nerves, stimulation was performed at the sciatic notch, stifle, and hock, with recording from the plantar interosseus muscles. Analysis included evaluation of compound muscle action potential amplitude (measured from largest negative to largest positive peak in mV), morphology, latency (measured in ms), and calculation of motor nerve conduction velocity (measured in m/s). Additional studies included late wave potentials (F‐waves, H‐reflex), cord dorsum potential, and repetitive nerve stimulation performed according to published protocols.^23^, ^24^, ^25^, ^26^, ^27^, ^28^ F‐waves and H‐reflex were recorded from the plantar interosseus muscles after distal tibial (hock) stimulation. F‐wave persistence, F‐wave minimum latency, F‐ratio, H‐reflex threshold, H‐reflex minimum latency, and H:M wave ratio were evaluated. Sensory nerve conduction was not evaluated. Cord dorsum potential was recorded from the L5‐L6 interarcuate space after distal tibial stimulation with the presence or absence and onset latency recorded. Repetitive nerve stimulation was performed by stimulation at the hock at 3 Hz, with recording from the plantar interosseus muscles. The amplitude of the first and fifth waveforms were compared to determine if there was a decrement (defined as >10% decrease) with repetitive stimulation. Electrophysiological data were interpreted using published reference values where available for cats and compared to results for cats in our database.^21^, ^24^, ^26^, ^28^

Biopsies were collected from the right triceps and cranial tibial muscles, and right common peroneal nerve by an open procedure under the same anesthetic episode as electrodiagnostic testing. Unfixed muscle biopsies were wrapped in a saline dampened gauze sponge and chilled, or immersion fixed in 10% neutral buffered formalin. All samples were sent by a courier service to the Comparative Neuromuscular Laboratory, University of California San Diego. Unfixed muscle specimens were flash frozen in isopentane precooled in liquid nitrogen and stored at −80°C until further processed by a standard panel of histochemical stains and reactions, including hematoxylin and eosin, modified Gomori trichrome, periodic acid Schiff, ATPases at pH 9.8 and 4.3, esterase, reduced nicotinamide adeine‐dinucleotide‐tetrazolium reductase (NADH‐TR), acid phosphatase, alkaline phosphatase, oil red O and Staphylococcal protein A‐Horseradish peroxidase (SPA‐HRPO).^29^ The fixed muscle biopsies were processed routinely into paraffin. A fixed biopsy from the common peroneal nerve was embedded in epoxy resin, cut into 1 μm thick sections, and stained with toluidine blue.

### 2.3 Pedigree analysis

Blood samples were obtained for DNA extraction from the 4 related affected Siberian cats and from 9 unaffected close relatives for future genetic studies (approved by IACUC, protocol #1812001840). All 13 cats were registered with either the The International Cat Association or the World Cat Federation. Pedigree information was obtained from all cats, and 1 large multigeneration pedigree, demonstrating familial relationships, was manually assembled and assessed for potential mode of inheritance. All cats were assigned an anonymized sample number for identification.

## 3 RESULTS

### 3.1 Signalment, history, and clinical signs

Thirteen related Siberian cats were included in the study, 4 clinically affected cats (3 female, 1 male) and 9 clinically unaffected control cats (5 female, 4 males). All cats originated from the same breeder and all affected cats were living in separate households at the time of the study. Unaffected control cats were all over 1 year of age when enrolled and were apparently healthy; none was reported to have had clinical signs of neuromuscular weakness or been diagnosed with a polyneuropathy. In the 4 affected cats, median age at onset of clinical signs was 8.5 months (range, 4‐10 months). Affected cats were reported to be normal, healthy kittens prior to the onset of signs, were indoor only, and had received routine vaccinations and been dewormed as kittens.

All 4 affected cats had histories of progressive or waxing and waning weakness consistent with neuromuscular disease. Prior to presentation to a veterinarian, signs were noted for 1 week or less in 3 cats and for 2 months in 1 cat. Owner complaints included difficulty jumping (3/4), abnormal gait (3/4), and reluctance to stand (1/4). Three of the 4 cats were evaluated both by a primary care veterinarian and veterinary neurologist. One cat was evaluated solely by a primary care veterinarian. Physical examination revealed paraparesis in 3 cats, and tetraparesis (more significant in the pelvic limbs) in 1 cat. Two cats displayed a plantigrade stance. Mentation, cranial nerve examination, and proprioception were normal in all 4 cats. Spinal reflex testing was documented for the 3 cats evaluated by veterinary neurologists. Patellar reflexes were normal in all 3 cats. Withdrawal reflexes were variably affected, with all cats showing decreased pelvic limb withdrawal reflexes and 2 cats showing decreased thoracic limb withdrawal reflexes. One cat also had a reduced perineal reflex and 1 cat was noted to have moderate thoracolumbar hyperesthesia. Clinical evidence of sensory or autonomic dysfunction was not noted in any cat.

### 3.2 Clinicopathological and imaging findings

Results of routine CBC and biochemical analyses were available for all affected cats. Results were within normal limits, with the exception of mild thrombocytopenia (147 000/L; reference range, 300 000‐800 000/L) in 1 cat and mild hyperphosphatemia (7.5 mg/dL; reference range, 2.9‐6.3 mg/dL) in another cat. Creatine kinase activities were available for 2 cats and both showed mild increases (625 UI/L, reference range, 64‐440 IU/L; 841 IU/L, reference range, 45‐695 IU/L). Results of feline immunodeficiency virus and feline leukemia virus serology were negative in the 3 cats in which testing was performed. One cat underwent additional testing including infectious disease titers, acetylcholine receptor antibody titer and cerebrospinal fluid analysis after lumbar puncture. Antibody testing was negative for feline coronavirus (7b protein antibody) and toxoplasma (IgM and IgG). The acetylcholine receptor antibody titer was within reference range. Cerebrospinal fluid analysis showed albuminocytologic disassociation with an elevated protein (94 mg/dL; reference range, <35 mg/dL), normal nucleated cell count (3 cells/μL; reference range, <5 cells/μL) and no abnormalities on cytologic evaluation.

Spinal radiographs were performed in 2 cats. No abnormalities were detected in either cat, with the exception of open vertebral growth plates in 1 cat considered an age‐appropriate finding.

### 3.3 Electrodiagnostic testing

**FIGURE 1:**
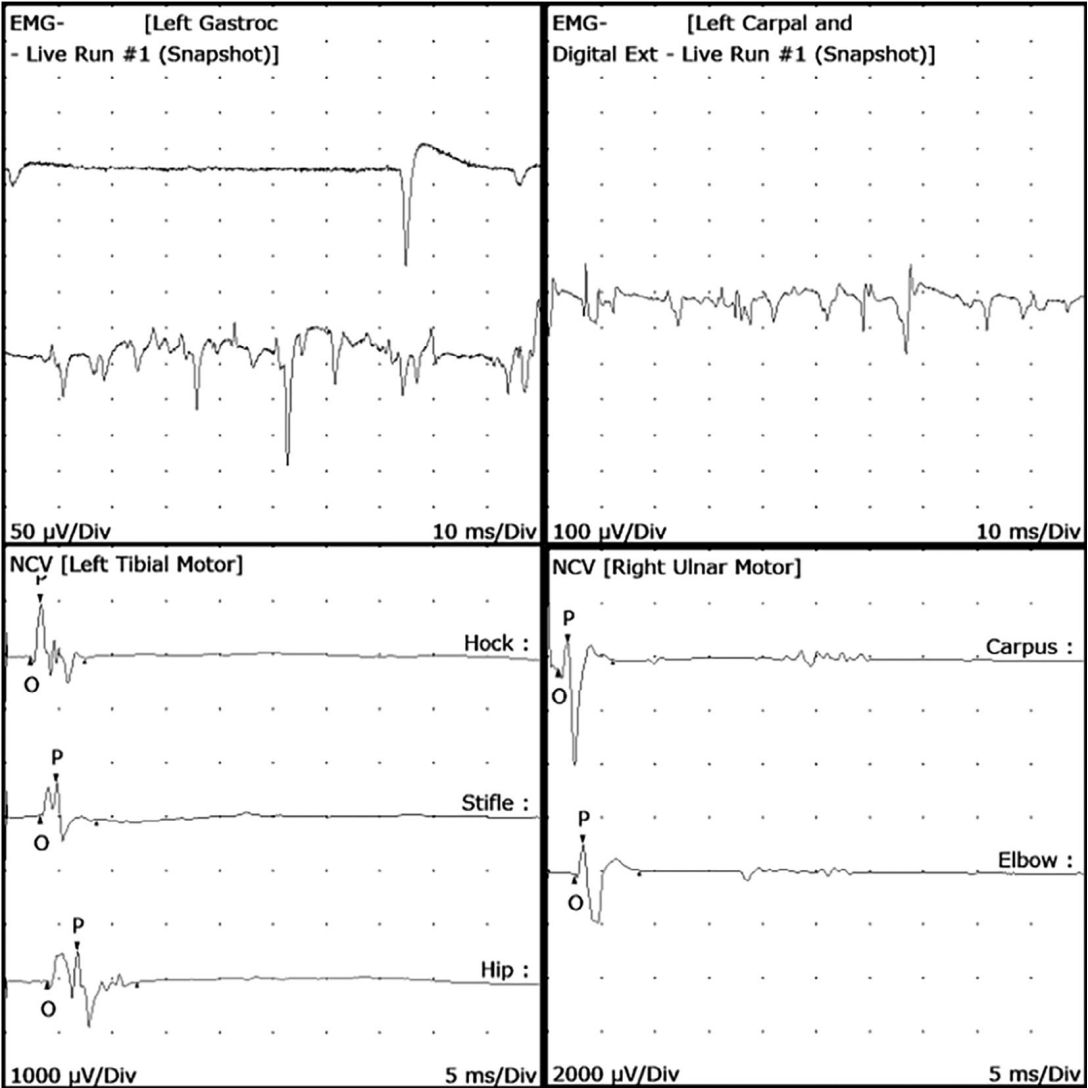
Electrodiagnostic testing results in 1 cat. Electromyography (top panels) demonstrated spontaneous activity in multiple muscles. Motor nerve conduction studies are depicted for the left sciatic/tibial nerve (bottom left) and right ulnar nerve (bottom right). There was normal conduction velocity, reduced compound muscle action potential amplitude and, for sciatic/tibial nerve, polyphasia

Electrodiagnostic testing was performed in 1 affected cat. Electromyography demonstrated moderate, multifocal spontaneous activity consisting of fibrillation potentials and positive sharp waves in multiple appendicular muscles (Figure 1). Electromyographic abnormalities varied from 1+ to 2+ (mild to moderate) according to a published grading scale.^19^ Changes were subjectively more severe in distal compared to proximal musculature. Changes were also more prominent in pelvic limb muscles than thoracic limb muscles, consistent with this cat's neurologic examination.

Motor nerve conduction studies of the ulnar and sciatic/tibial nerves were abnormal (Figure 1). For the sciatic/tibial nerves, compound muscle action potential peak to peak amplitude was reduced (hock: 1.6 mV, stifle: 1.2 mV, hip: 1.5 mV) and there was moderate polyphasia and temporal dispersion. For the ulnar nerve, compound muscle action potential amplitude was decreased (carpus: 4.3 mV, elbow: 3.0 mV), but polyphasia was minimal. Motor nerve conduction velocity was normal for both ulnar and sciatic/tibial nerves (sciatic/tibial: 82 m/s, ulnar: 74 m/s). With distal tibial stimulation, minimum F‐wave latency was 13 ms (calculated expected latency was 7.4 ms), F‐wave persistence was 80% and F ratio was 1.9. H‐reflex was present with a threshold intensity of 11 mA, minimum latency of 9 ms, and H : M ratio of 10%. Repetitive nerve stimulation was normal with no decrement and cord dorsum potential was normal.

### 3.4 Histopathology

**FIGURE 2:**
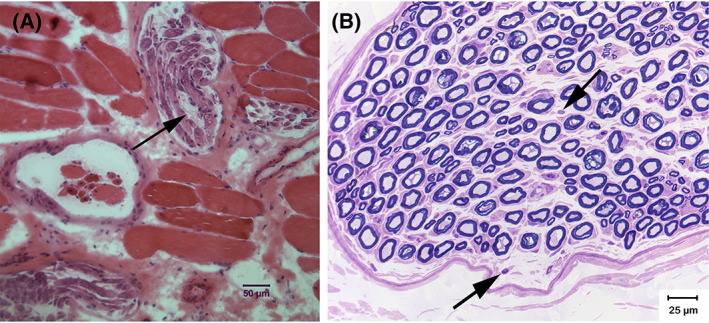
Histopathology of muscle and nerve biopsy samples in 1 affected cat. A, H&E stained cryosection from the triceps muscle. Intramuscular nerve branches are shown among groups of mildly atrophic myofibers. Mild pathological changes including endoneurial edema (arrow) and mild nerve fiber loss are noted within intramuscular nerve branches. A normal appearing muscle spindle is on the midleft side of the image. B, Resin embedded section of the common peroneal nerve. The density of myelinated fibers is subjectively appropriate without obvious axonal degeneration or demyelination. Numerous collagenous bundles consistent with resolving subperineurial and endoneurial edema and minimal mononuclear cell infiltrates (highlighted by arrows) were noted

Histopathology of right cranial tibial muscle, right triceps muscle, and right common peroneal nerve were available for 1 affected cat. A mild variability in myofiber size was present in both muscles with scattered atrophic fibers having an anguloid shape. Fiber type grouping was not observed. Intramuscular nerve branches showed moderately severe edema, mild regional depletion of myelinated nerve fibers, and mild scattered mononuclear cell infiltrates (Figure 2A). Nerve biopsy showed subjectively normal nerve fiber density with numerous collagenous bundles in the subperineurium and endoneurium consistent with resolving edema (Figure 2B). Neither axonal nor myelin abnormalities were identified. Minimal mononuclear cell infiltrations were present in the endoneurium with some proximity to nerve fibers (Figure 2B). The changes in the intramuscular nerve branches and common peroneal nerve were consistent with an early or mild predominantly distal polyneuropathy with associated inflammation.

### 3.5 Treatment and follow‐up

All 4 affected cats were initially treated with robenacoxib using the labeled dose recommendations based on body weight (Onsior, Elanco Animal Health, Greenfield, Indiana). One cat was administered a single subcutaneous injection of robenacoxib. One cat was administered a subcutaneous injection, followed by 3‐day course of robenacoxib PO. The remaining 2 cats were treated with a 3‐day course of robenacoxib PO. Two cats showed improvement when treated with robenacoxib; 1 normalized within days of initiation while the speed of improvement could not be confirmed in the other cat. Two cats showed no improvement and were switched to a tapering course of prednisolone (initial dose 1 mg/kg/day and 1.5 mg/kg/day PO). Regardless of treatment, all 4 cats were clinically normal within 1 month of initial presentation.

All 4 cats had at least 1 documented relapse of their neuromuscular weakness. In 3 cats, signs of relapse occurred within 1 to 3 months of the initial episode while relapse was noted 9 months later in the fourth cat. Two of these relapses were successfully managed with robenacoxib PO. The remaining 2 cats were both still on a tapering course of prednisolone at the time of recurrence of weakness; signs resolved when the prednisolone dose was transiently increased. Upon follow‐up with the owners of the affected cats, 3 cats were reported to be neurologically normal 1 to 2 years after their initial presentation with no further episodes of weakness. One cat (whose first relapse occurred at 3 months after initial signs of weakness) was reported to have had 2 additional episodes of mild weakness characterized by being less active and mild difficulty jumping. These were noted by the owner at approximately 6 months and 20 months after the initial episode. The cat was not evaluated by a veterinarian during these additional occurrences, no treatment was instituted, and signs resolved gradually over several months. At the time of writing, this cat was reported to be normal at home with no residual weakness.

### 3.6 Pedigree analysis

**FIGURE 3:**
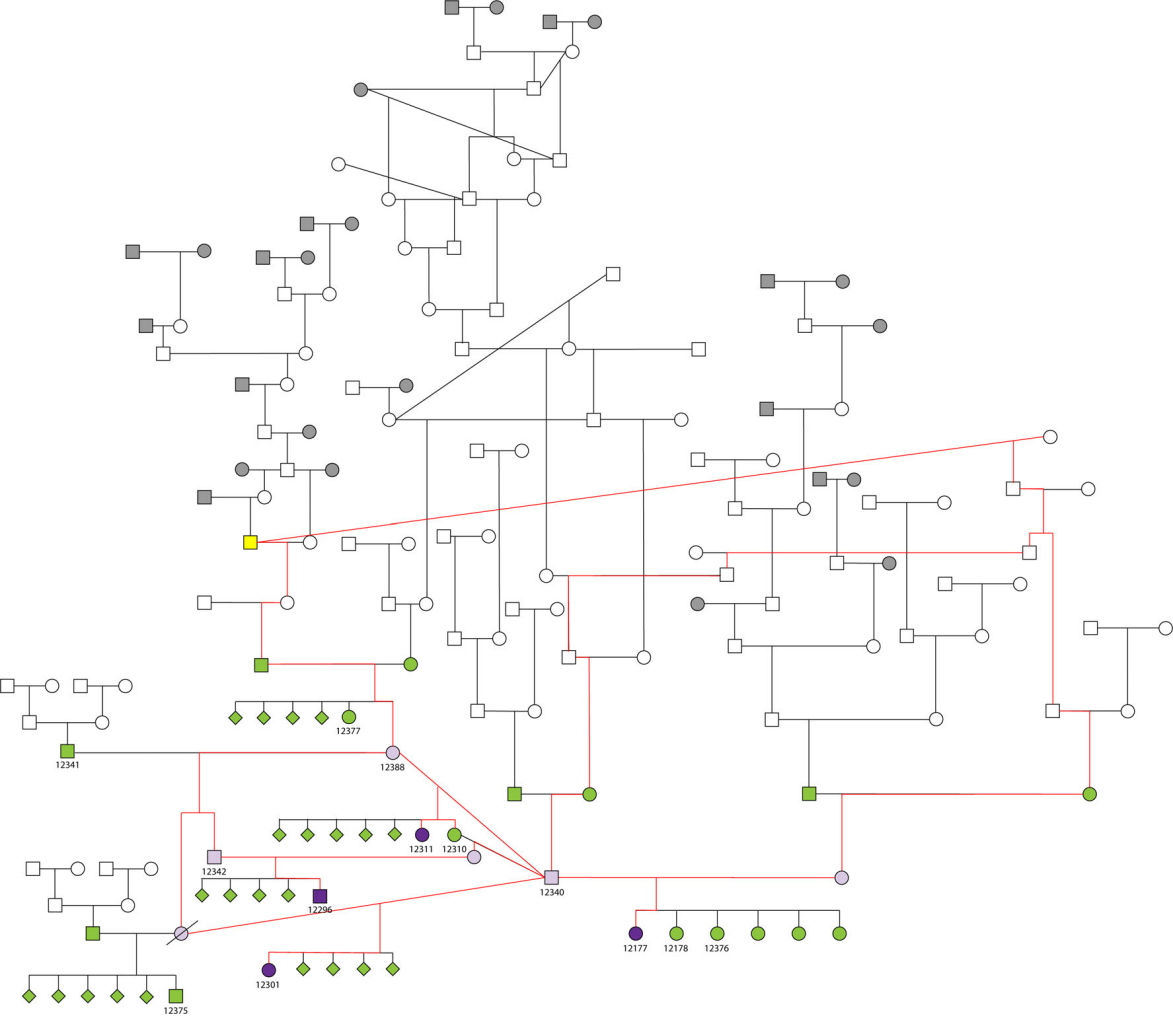
Pedigree of affected Siberian Forest cat family. The 13 cats in the present study are each indicated with a 5 digit sample number. The 4 affected cases are filled in with dark purple and those shaded a light purple are obligate heterozygotes (under an autosomal recessive mode of inheritance); in the latter group, if a sample number is provided, that animal is confirmed unaffected, whereas the remainder are presumed unaffected (no reported neurologic abnormalities). Individuals with a sample number and filled in green are confirmed controls, whereas those filled in green without a sample number are reported controls but without direct owner or veterinary confirmation. Symbols filled in with gray are founder Siberian cats. For both gray and nonfilled symbols (ie, white), no health information was available. The red lines demonstrate pathways tracing all affected cats and obligate carries to a common relative (sire), which is shaded yellow. Round symbols = females, squares = males, diamonds = unknown sex, and “/” indicates known deceased animal. Note: for clarity, not all relationships are shown

All affected cats were closely related; specifically, 3 were half‐siblings (#12177, 12301, and 12311; sharing the same sire) and the fourth's (#12311) mother was also produced by that same sire. Cat #12177 underwent the thorough diagnostic evaluation for neuromuscular disease as outlined above. All 9 control cats (#12178, 12310, 12340, 12341, 12342, 12375, 12376, 12377, and 12388) were healthy with no history of neuromuscular weakness and were also closely related to the cases. Control status was confirmed for these 9 cats through owner‐supplied answers to a health questionnaire. Other related cats were reported as clinically normal via the health questionnaires completed by the owners of the affected or control cats who knew that the remainder of the litter and parents were healthy. Therefore, they were presumed to be unaffected but specific confirmation of health status was not available for these additional cats. A pedigree was assembled (Figure 3), demonstrating these relationships; inbreeding is observed, a not uncommon feature among purebred cats. All obligate carrier cats can be traced back to a common ancestor sire within 3 to 6 generations. The pedigree is highly suggestive of a single‐gene (Mendelian), autosomal recessive mode of inheritance, although a genetically complex/polygenic condition cannot be ruled‐out. A sex‐linked condition is not suspected due to having both male and female affected cats.

## 4 DISCUSSION

We described clinical features of a novel motor polyneuropathy in juvenile Siberian cats characterized by self‐limiting neuromuscular weakness. An inherited polyneuropathy or inherited predisposing gene variant is suspected with a possible autosomal recessive mode of inheritance, but other potential underlying causes including a nongenetic inflammatory etiology have not been eliminated. While outcome was good in affected cats, relapse was common, especially within the first several months of diagnosis.

This was the first report of a polyneuropathy in the Siberian breed of domestic cat. Overall polyneuropathies are relatively uncommon in cats and are more commonly associated with a generalized syndrome affecting both the central and peripheral nervous systems.^1^, ^2^, ^3^, ^13^ Our findings demonstrated clinical signs in juvenile cats less than a year of age with an acute to subacute onset. In prior reports involving multiple breeds, age of onset is similar while duration of signs prior to presentation is typically longer (weeks to months rather than days to weeks) with many cats showing recurrent or progressive signs in the months prior to presentation.^14^, ^16^, ^18^ It is possible that the cats presented here would have demonstrated a similar course but that fortuitous identification of 2 potentially related cats showing similar signs of weakness, together with contact with the breeder, led to more rapid veterinary evaluation in other related cats.

All 4 affected cats showed progressive or waxing and waning neuromuscular weakness of variable severity without overt clinical evidence of sensory or autonomic dysfunction. Neurologic examination abnormalities were also confined to appendicular weakness with no cranial nerve involvement. While other reports of juvenile polyneuropathies in cats describe similar presenting complaints of weakness and gait abnormalities, often worse in the pelvic limbs, there are notable distinctions.^14^, ^16^, ^18^ In the largest case series to date describing 37 Bengal cats with signs of polyneuropathy, reported abnormalities in addition to limb weakness include 8 cats with cranial nerve deficits, 3 with altered pain perception, 14 with hyperesthesia, and muscle atrophy in 18 cats.^14^ One cat in our cohort demonstrated spinal hyperesethesia but otherwise none of these clinical signs were apparent in our cases. Similarly, in a report of 5 young cats of different breeds with a motor polyneuropathy, 3 cats show generalized muscle atrophy and 3 also have cranial nerve deficits including 1 cat each with a decreased menace response, decreased palpebral reflex, and decreased gag reflex.^18^ In a report of 2 Snowshoe cats, other neurologic abnormalities include ataxia and mild vestibulocerebellar deficits, both suggestive of possible concurrent central nervous system involvement.^17^ Three of the 4 cats in the present study were evaluated by veterinary neurologists, so it is possible that subtle deficits were present but not identified in the other cat in which a thorough neurologic examination could not be confirmed.

Electromyography and motor nerve conduction study results in the 1 cat that underwent thorough neuromuscular evaluation were consistent with a motor polyneuropathy with evidence of axonal dysfunction, possible mild or secondary demyelination, and a distal distribution of disease. Histologically, this was supported by nerve fiber loss within the distal intramuscular nerve branches. However, the biopsy lacked obvious changes consistent with a primary axonal or myelin‐associated neuropathy. Other abnormalities in the distal intramuscular nerve branches included edema and mononuclear cell infiltration; minimal mononuclear cell infiltration was also noted in the peroneal nerve. These changes suggested an inflammatory component. This was distinct from the histopathological changes in the young cats with a motor polyneuropathy and the chronic relapsing polyneuropathy in young Bengal cats.^14^, ^18^ The latter is characterized by demyelination and remyelination with inappropriately thin myelinated fibers and supernumerary Schwann cells (presumptive onion‐bulb formations) in the peripheral nerves.^14^ For future study, additional samples from other affected Siberian cats and more in‐depth evaluation of myelin including teased nerve fibers and electron microscopy should be pursued.

Albuminocytologic dissociation was also noted in the cat that had more extensive diagnostic testing. This finding indicated involvement of the proximal nerve and breakdown of the blood: nerve barrier in the subarachoid space of ventral nerve roots.^30^ While F‐waves and H‐reflex can aid in electrodiagnostic evaluation of the proximal nerve, there is limited published information available for cats. Compared to published data obtained from normal cats,^28^ the cat in this report had a longer F‐wave latency than expected and decreased F‐wave persistence, suggesting involvement of the proximal nerve segment or ventral nerve root. However, the H‐reflexes appeared normal and the F ratio was normal compared to what is reported for dogs, which could indicate equal distribution of disease between the proximal and distal nerve segments.^25^, ^26^, ^31^, ^32^ Because biopsies of the ventral nerve root or proximal nerve are not routinely performed, the exact distribution of disease including the degree and nature of proximal involvement of the nerve was unclear.

The clinical outcome was positive in all 4 cases, though relapse was common. Three cats had a single relapse that occurred between 1 and 9 months after diagnosis. These cats recovered fully and none went on to have subsequent recurrence of weakness. One cat had a total of 3 relapses over a period of approximately 1 and half years, including 2 self‐limiting episodes of weakness described by the owner. At the time of writing this report, all cats were still alive without additional relapses. This disease course was distinct from the chronic, relapsing course reported in the Bengal cats, where there are typically multiple relapse events with clear evidence of repeated demyelination and subsequent remyelination on biopsy samples.^14^ Follow‐up information in other reports suggests improvement or stabilization of weakness occurs in the majority of cases but relapses of varying severity are common.^17^, ^18^

It was not possible to determine if treatments administered had an effect on duration of signs or outcome. Subjectively, 2 cats appeared to improve rapidly upon starting robenacoxib and 1 of those cats seemingly responded favorably again after a relapse. However, the other 2 cats showed no response and were transitioned to prednisolone. It was similarly difficult to determine if prednisolone positively altered the course of disease. These 2 cats did suffer relapses when prednisolone was tapered and improved when the dose was transiently increased; however, 1 of these cats had subsequent episodes of mild weakness that resolved without any therapy. It is, therefore, possible that the disease course and relapses were independent of treatment and that the cats might have improved spontaneously over time. The need or benefit of specific treatment remains unknown in the Siberian cats. Spontaneous improvement of weakness is reported in 2 young Snowshoe cats over the course of 1 to 2 years and in an Abyssinian kitten with polyneuritis within weeks of diagnosis.^16^, ^17^ The majority of cats in published reports are variably treated with nonsteroidal or steroid anti‐inflammatory medications, among other medications, resulting in similar inability to determine any benefit of specific therapy.^14^, ^15^, ^16^, ^18^ Further studies are warranted to explore the likelihood of spontaneous remission or, specifically, if a short course of a nonsteroidal anti‐inflammatory alters time to remission.

Determination of an underlying cause of polyneuropathy can be challenging. Diagnostic tests performed varied between cats and only 1 underwent a thorough neuromuscular disease evaluation. Based on available diagnostic information, acquired causes such as obvious infection, intoxication, metabolic disease, or neoplasia were considered unlikely. While neuromuscular weakness is an uncommon presentation overall, immune‐mediated polyradiculoneuritis is relatively frequently identified as a cause in young cats.^1^, ^6^, ^8^, ^9^, ^11^, ^16^ Therefore, acquired idiopathic or immune‐mediated polyradiculoneuritis or polyneuritis were considered possible and explain some of the biopsy, electrodiagnostic, and cerebrospinal fluid analysis findings. Albuminocytologic dissociation is reported with inflammatory conditions, including Guillain‐Barre syndrome in people and acute polyradiculoneuritis in dogs and cats.^9^, ^30^, ^33^, ^34^ Additionally, biopsy samples in the affected cat reported here demonstrated mild mononuclear cell infiltration consistent with an inflammatory cause but could not confirm if such inflammation was present at the level of the ventral nerve roots, as would be expected with polyradiculoneuritis. Acute polyradiculoneuritis presents suddenly with rapid progression from paraparesis to tetraparesis or tetraplegia, often with respiratory fatigue.^30^, ^33^ While 1 of our cases displayed mild thoracic limb paresis, the remainder were paraparetic and electrodiagnostic findings were not clearly convincing for polyradiculoneuritis. Importantly, albuminocytologic dissociation is also reported with other idiopathic and inflammatory conditions including chronic inflammatory demyelinating polyneuropathy,^4^, ^34^, ^35^, ^36^ idiopathic vestibular and facial neuropathy^37^ as well as in 6 of 13 Bengal cats with chronic relapsing polyneuropathy.^14^

There were overlapping clinical and histological features with a motor polyneuropathy reported in 5 young, unrelated cats of multiple breeds where 1 or more acquired conditions are suspected.^18^ It remains possible that not all cats in the present study were suffering from the same disease and that, as stated above, acquired conditions such as a nongenetic inflammatory polyneuropathy could cause similar, nonspecific histopathologic changes. Injury to peripheral nerves regardless of the cause results in a limited number of pathological changes and it is difficult to predict an inciting cause. However, age of onset, clinical presentation, and disease course were strikingly similar in this cohort, despite each case living in separate homes, supporting diagnosis of the same motor polyneuropathy in all 4 cats.

Because this is a group of closely‐related pure‐bred cats, a shared, inherited condition was also considered a plausible potential etiology. Given the histologic findings, variant genes might be associated with mediators of inflammation or predisposing factors to immune dysfunction and not necessarily those directly involving axonal or myelin function as have been suspected or demonstrated in other canine and feline inherited polyneuropathies.^1^, ^2^, ^38^, ^39^, ^40^, ^41^, ^42^, ^43^, ^44^ Such a scenario is hypothesized in Siberian Huskies with a presumed inherited polyneuropathy, as some dogs show prominent inflammatory changes on histopathology.^45^ Further, in Newfoundlands with an early‐onset immune‐mediated Myasthenia Gravis, a genotype association exists within the canine major histocompatibility complex, specifically the dog leukocyte antigen class I.^44^ However, a P_0_ (myelin structural protein in the peripheral nervous system) knockout mouse model produces a neuropathy resembling chronic inflammatory demyelinating polyneuropathy.^46^ This raises the possibility that some patients with chronic inflammatory demyelinating polyneuropathy might actually have an underlying inherited demyelinating condition that somehow triggers an inflammatory component. While pedigree analysis of this condition supported an autosomal recessive mode of inheritance, a polygenic trait remains possible. If a specific single causative gene mutation is not identified, then a genetic predisposition (polygenic inheritance) would be considered. Genome‐wide investigation is required under either inheritance model (single‐gene versus polygenic), giving particular consideration to genes involved in the nervous and immune systems, as well as those influencing inflammation and local tissue environments. Polygenic inheritance is now suspected in the Bengal cats, for which a genetic cause of their relapsing polyneuropathy has not yet been reported. Whole genome sequencing is ongoing in this cohort using submitted DNA samples. Elucidation of a specific etiology, genetic or otherwise, could help to guide breeding decisions for Siberian breeders. It might also shed light on development of motor polyneuropathies in other cats, including presumed idiopathic or immune‐mediated acquired forms.

The 9 control cats were not specifically evaluated by a veterinarian at the time of data collection to confirm their clinical health status, which was instead reported directly by their owners. Given the severity of the condition in affected cats, it seemed unlikely that any of the control cats' owners would have missed this clinical syndrome in their pet. We also lacked extensive health information for additional “unaffected” littermates and parents of the affected cats that were used when constructing the pedigree. However, these were typically described as “normal” by the breeder or the owner of affected or control cats mentioning that the remainder of the kittens in the litter were normal. We feel it is unlikely that any of these cats were misclassified given the young age of onset and overt nature of the weakness displayed by affected cats.

## CONFLICT OF INTEREST DECLARATION

Authors declare no conflict of interest.

## OFF‐LABEL ANTIMICROBIAL DECLARATION

Authors declare no off‐label use of antimicrobials.

## INSTITUTIONAL ANIMAL CARE AND USE COMMITTEE (IACUC) OR OTHER APPROVAL DECLARATION

Approved by the Purdue University IACUC, protocol #1812001840.

## HUMAN ETHICS APPROVAL DECLARATION

Authors declare human ethics approval was not needed for this study.
